# High-dose steroid therapy for idiopathic optic perineuritis: a case series

**DOI:** 10.1186/1752-1947-4-404

**Published:** 2010-12-10

**Authors:** Maria Tatsugawa, Hidetaka Noma, Tatsuya Mimura, Hideharu Funatsu

**Affiliations:** 1 Department of Ophthalmology, Hiroshima Prefectural Hospital, Hiroshima, Japan, 1-5-54, Ujinakanda, Minami-ku, Hiroshima 734-8530, Japan; 2 Department of Ophthalmology, Yachiyo Medical Center, Tokyo Women's Medical University, 477-96, Owada-shinden, Yachiyo, Chiba 276-8524, Japan; 3 Department of Ophthalmology, University of Tokyo Graduate School of Medicine, 7-3-1, Hongo, Bunkyo-ku, Tokyo 113-0033, Japan

## Abstract

**Introduction:**

It has been reported that the prognosis of optic perineuritis may be poor when initiation of treatment is delayed. Here we report the successful treatment of three patients with idiopathic optic perineuritis, including two in whom initiation of therapy was delayed.

**Case presentation:**

Three Japanese patients (two women aged 73 and 66 years, and one man aged 27 years) presented with loss of vision (for five months, several months, and two months respectively) and pain on eye movement in the third case only, and were diagnosed as having idiopathic optic perineuritis. Fat-suppressed T2-weighted magnetic resonance images showed high signal intensity areas around the affected optic nerves, suggesting the presence of optic perineuritis. Two patients received steroid pulse therapy and the third was given high-dose steroid therapy. The visual acuity improved in all three cases.

**Conclusion:**

High-dose steroid therapy may be effective for idiopathic perineuritis in patients without optic nerve atrophy, even if initial treatment (including moderate-dose steroids) has failed.

## Introduction

Idiopathic optic perineuritis has been reported as a type of orbital inflammatory pseudotumor [1-3]. Currently, the diagnosis of optic perineuritis is most commonly based on magnetic resonance image (MRI) findings along with the clinical characteristics. Although some reported cases have been diagnosed by pathologic examinations, the distinction between optic neuritis and optic perineuritis is generally radiographic [4]. The characteristic differences between idiopathic optic perineuritis and idiopathic optic neuritis are as follows [5]: The age distribution of the former is wide and it particularly affects elderly patients, and a paracentral scotoma or an arcuate defect are frequent findings. The onset is slow (usually over several weeks), and recovery is often poor in patients with optic perineuritis when treatment is delayed. The response to corticosteroids is often dramatic, although recurrence is common with tapering of therapy. Here, we report the successful treatment of three patients with idiopathic optic perineuritis who received high-dose steroid therapy.

## Case presentations

### Case 1

**Figure 1:**
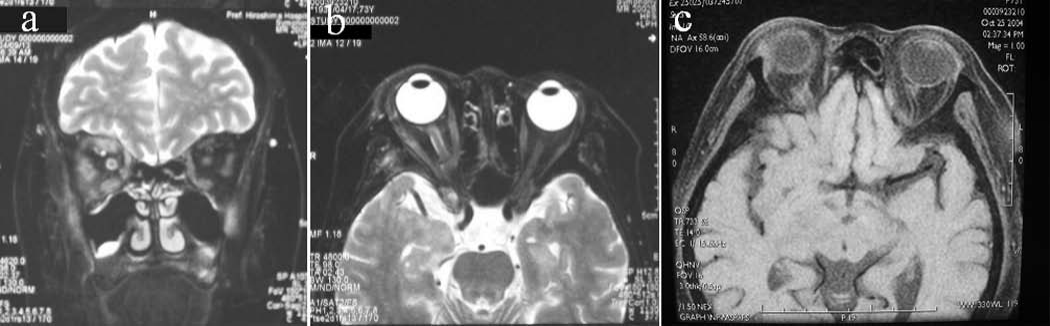
**Fat-suppressed T2-weighted MR images of Case 1**. Coronal image (a) and axial image (b) from a 73-year-old Japanese woman with idiopathic optic perineuritis. There is a high signal intensity area around the right optic nerve and moderate swelling of the right extraocular muscles, suggesting inflammation around the optic nerve sheath and the extraocular muscles. Fat-suppressed T1-weighted magnetic resonance image obtained one month after steroid pulse therapy. This axial image (c) shows persistence of the high signal intensity area around the right optic nerve and moderate swelling of the right extraocular muscles. The extraocular muscles showed persistent moderate swelling (not visible on this image).

A 73-year-old Japanese woman had noticed a decrease in vision in her right eye for five months. On examination, the visual acuity on her right side was 20/60. She was treated with prednisolone at doses of up to 30 mg/day for eyelid swelling. After five months, however, her acuity was only 20/400 on her right side. A right relative afferent pupillary defect was present. Goldmann perimetry showed an arcuate scotoma of her right eye. Laboratory tests revealed a CRP of 0.9 mg/dl (normal range: 0.0-0.3) and an ESR of 38 mm/hour, while ACE, FTA, and ANCA were all within the normal range. The results of hematology tests, renal and liver function tests, urine analysis, chest radiography, and computed tomography were all within normal limits. Fat-suppressed T2-weighted MR images revealed a high signal intensity area around her right optic nerve and moderate swelling of her right extraocular muscles, suggesting inflammation of her optic nerve sheaths and extraocular muscles (Figure 1). Steroid pulse therapy was initiated. After four days, the vision of her right eye improved to 20/80. One month after steroid pulse therapy, fat-suppressed T1-weighted MR images showed persistence of the high signal intensity area around her right optic nerve and moderate swelling of her right extraocular muscles (Figure 1). Subsequently, the steroid dose was gradually tapered. There has been no recurrence of symptoms after an observation period of 22 months.

### Case 2

**Figure 2:**
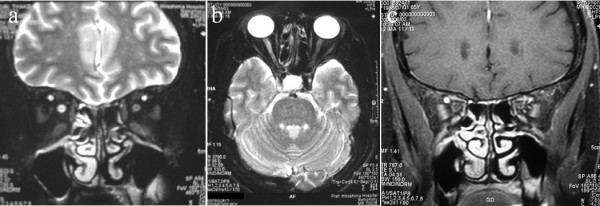
**Fat-suppressed T2-weighted images of Case 2**. Coronal image (a) and axial image (b) from a 66-year-old Japanese woman show high-intensity areas in the right optic nerve sheath. Fat-suppressed T1-weighted post-contrast coronal image (c) shows high-intensity areas in the right optic nerve sheath. The optic nerve sheath is enlarged and enhanced on both sides.

A 66-year-old Japanese woman presented to our hospital with decreased vision in her right eye that had persisted for several months. Vision was 20/300 on her right side and a relative afferent pupillary defect was detected, although there were no abnormal intraocular findings. Goldmann perimetry showed an arcuate scotoma of her right eye. Laboratory tests revealed a CRP of 0.3 mg/dl, while the ESR, ACE, FTA, and ANCA were all within the normal range. The results of hematology tests, renal and liver function tests, urine analysis, chest radiography, and computed tomography were all within normal limits. MR images showed high-intensity areas in her right optic nerve sheath on fat-suppressed T2-weighted images and fat-suppressed T1-weighted images (Figure 2); these findings suggesting inflammation of her optic nerve sheath. Treatment with prednisolone (40 mg/day) was initiated. Subsequently, the steroid dose was gradually tapered. After two months, there was a recurrence of symptoms in her right eye, so prednisolone (40 mg/day) was started again. Subsequently, the steroid dose was tapered more gradually and her vision was 20/20 on the right side after 11 months. Recurrence of symptoms has not been detected after follow-up for 19 months.

### Case 3

**Figure 3:**
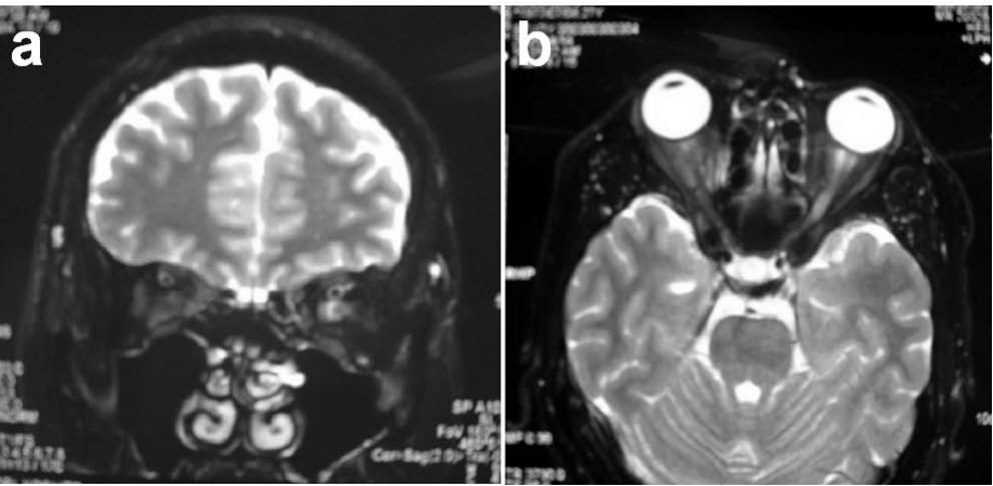
**Magnetic resonance images of Case 3**. T2-weighted coronal (a) and axial (b) images demonstrate strong hyperintensity around the left optic nerve of a 27-year-old Japanese man.

A 27-year-old Japanese man came to our hospital with blurred vision in the upper field of his left eye and ocular pain/headache associated with eye movement that had persisted for two months. His visual acuity was 20/300 on his left side. There was swelling and erythema of his left optic disc. Goldmann perimetry showed enlargement of Mariotte's blind spot and a paracentral scotoma of his left eye. Laboratory tests revealed a CRP of 0.5 mg/dl, while the ESR, ACE, FTA, and ANCA were all within the normal range. The results of hematology tests, renal and liver function tests, urine analysis, chest radiography, and computed tomography were all within normal limits. MR images revealed no abnormalities in his brain. On fat-suppressed T2-weighted images, the area around his left optic nerve showed a high intensity (Figure 3). Steroid pulse therapy was initiated. After seven days, his vision improved to 20/15 on the left. Subsequently, the dose of steroids was gradually reduced. No recurrence has been noted after 15 months.

## Discussion

The prognosis of optic perineuritis has been reported to be poor when initiation of treatment is delayed [5]. However, our first two cases both responded well to steroid therapy and achieved a good visual prognosis, despite the interval between the onset of symptoms and initiation of treatment being longer than six months. Concerning the steroid dose, recurrence was observed in Case 2 after treatment with prednisolone at a daily dose of 40 mg in the early stage of her illness, and Case 1 showed recurrence after receiving prednisolone at a dose of 30 mg/day at her previous hospital. After we performed steroid pulse therapy at our hospital for Cases 1 and 3, there was no recurrence in Case 3, and no subsequent recurrence in Case 1. Purvin *et al*. reported recurrence of optic perineuritis in 4 out of 14 patients treated with oral steroids at doses of 60-80 mg/day [5].

Perimetry was performed up to isopter I-1e in all of our patients. The innermost isopter of the central visual field that showed a response was isopter I-4e in Case 1, isopter I-2e in Case 2, and isopter I-1e in Case 3. Thus, Cases 1 and 2 did not respond to isopter I-1e, suggesting the presence of central depression. Perimetry of the peripheral visual fields including the paracentral field revealed arcuate constriction on the downside of isopter V-4e in Case 1, who showed a generalized decrease of sensitivity. In Case 2, depression was seen on the upside of Mariotte's blind spot in isopter I-4e, indicating a paracentral scotoma. In Case 3, scotomata were observed at three sites on the upside of Mariotte's blind spot in isopter III-4e. These results suggest that reduced vision was at least partly ascribable to a decrease of central visual field sensitivity in Cases 1 and 2, whereas vision was reduced despite the lack of a central scotoma or reduction of central field sensitivity in Case 3. Therefore, the reduced visual acuity was related to central or generalized depression of vision due to optic perineuritis in Cases 1 and 2. In Case 3, vision may have been reduced because scotomata involved the fixation point.

This case series had the following limitation. The best diagnostic sequence for optic perineuritis is post-contrast fat-suppressed T1-weighted images. On other images, the area of hyperintensity around the optic nerve could represent an increase of cerebrospinal fluid that would occur if there was optic atrophy. However, we did not obtain fat-suppressed T1-weighted images in all three cases, although such images are required for the definite diagnosis of optic perineuritis.

## Conclusions

Two patients with optic perineuritis who underwent steroid pulse therapy showed no recurrence, as did one patient receiving high-dose prednisolone. Our results suggest that steroid pulse therapy or high-dose prednisolone may be effective for idiopathic optic perineuritis in patients without optic nerve atrophy, even if initial treatment (including moderate-dose steroids) has failed.

## Abbreviations

ACE: angiotensin-converting enzyme; ANCA: anti-neutrophilic cytoplasmic antibodies; CRP: C-reactive protein; ESR: erythrocyte sedimentation rate; FTA: fluorescent treponemal-antibody; MR: magnetic resonance;

## Consent

Written informed consent was obtained from the patients for publication of this case series and any accompanying images. Copies of the written consents are available for review by the Editor-in-Chief of this journal.

## Competing interests

The authors declare that they have no competing interests.

## Authors' contributions

MT, HN, TM and HF analyzed and interpreted the patient data. MT was a major contributor in writing the manuscript. All authors read and approved the final manuscript.
